# Supplementary material for: A Novel Cuticular Protein-like Cpr21L Is Essential for Nymph Survival and Male Fecundity in the Brown Planthopper
Source: Int J Mol Sci. 2023 Jan 21;24(3):2163. doi: 10.3390/ijms24032163 (PMC9916611; doi:10.3390/ijms24032163)
Supplement: Supplementary file 1 [file ijms-24-02163-s001.zip › ijms-2123756-supplementary.pdf]

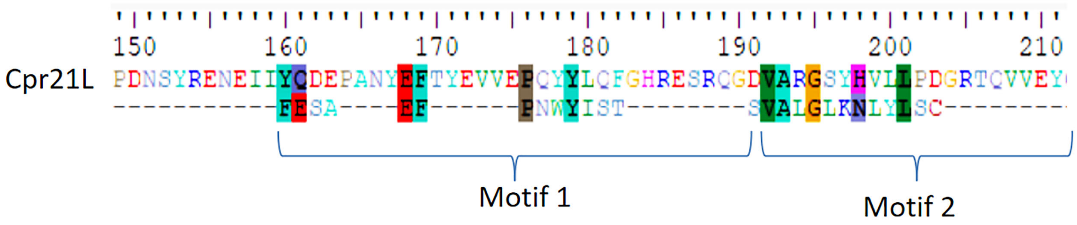

Figure S1. Alignment of Cpr21L with two motifs (motif-1 and motif-2). Two potential motifs, motif-1 and motif-2, are predicted by MEME based on the sequence of 15 cargoes the secretion of which was regulated by TMED10.
